# Artificial Intelligence and Machine Learning in Lung Cancer: Advances in Imaging, Detection, and Prognosis

**DOI:** 10.3390/cancers17243985

**Published:** 2025-12-14

**Authors:** Mohammad Farhan Arshad, Adiba Tabassum Chowdhury, Zain Sharif, Md. Sakib Bin Islam, Md. Shaheenur Islam Sumon, Amshiya Mohammedkasim, Muhammad E. H. Chowdhury, Shona Pedersen

**Affiliations:** 1 Department of Basic Medical Sciences, College of Medicine, QU Health, Qatar University, Doha 2713, Qatar; mi2307777@qu.edu.qa (M.F.A.); zs2309681@qu.edu.qa (Z.S.); sakib.binislam@qu.edu.qa (M.S.B.I.); 2 Department of Electrical and Electronics Engineering, University of Dhaka, Dhaka 1000, Bangladesh; adibatabassum-2016115013@eee.du.ac.bd; 3 Department of Electrical Engineering, Qatar University, Doha 2713, Qatar; sumon@qu.edu.qa (M.S.I.S.); amshiyamohammedkasim@gmail.com (A.M.)

**Keywords:** lung cancer, artificial intelligence, machine learning, radiomics and prognosis, deep learning in imaging

## Abstract

Background/Objectives: As the primary cause of cancer-related death globally, lung cancer highlights the critical need for early identification, precise staging, and individualized treatment planning. By enabling automated diagnosis, staging, and prognostic evaluation, recent developments in artificial intelligence (AI) and machine learning (ML) have completely changed the treatment of lung cancer. The goal of this narrative review is to compile the most recent data on uses of AI and ML throughout the lung cancer care continuum. Methods: A comprehensive literature search was conducted across major scientific databases to identify peer-reviewed studies focused on AI-based imaging, detection, and prognostic modeling in lung cancer. Studies were categorized into three thematic domains: (1) detection and screening, (2) staging and diagnosis, and (3) risk prediction and prognosis. Results: Convolutional neural networks (CNNs), in particular, have shown significant sensitivity and specificity in nodule recognition, segmentation, and false-positive reduction. Radiomics-based models and other multimodal frameworks combining imaging and clinical data have great promise for forecasting treatment outcomes and survival rates. The accuracy of non-small-cell lung cancer (NSCLC) staging, lymph node evaluation, and malignancy classification were regularly improved by AI algorithms, frequently matching or exceeding radiologist performance. Conclusions: There are still issues with data heterogeneity, interpretability, repeatability, and clinical acceptability despite significant advancements. Standardized datasets, ethical AI implementation, and transparent model evaluation should be the top priorities for future initiatives. AI and ML have revolutionary potential for intelligent, personalized, and real-time lung cancer treatment by connecting computational innovation with precision oncology.

## Simple Summary

Lung cancer remains the top cause of cancer death, and clinicians must interpret large, complex images and patient data quickly and consistently. This narrative review was undertaken to assess how artificial intelligence and machine learning can support earlier detection, more accurate diagnosis and staging, and clearer estimates of patient outcomes. We examined recent methods for finding and characterizing lung nodules, segmenting tumors, and predicting survival or treatment response, and we compare these tools with routine clinical practice. We also identify the main barriers to real-world use, including data differences across hospitals, limited transparency of algorithms, and the need for external validation. By mapping what works, where it fails, and how to evaluate it, our review aims to guide researchers toward robust, clinically useful models that accelerate safe adoption in precision lung-cancer care.

## 1. Introduction

Lung cancer remains the leading cause of cancer mortality worldwide, accounting for about one in five cancer deaths, largely because most cases are detected at an advanced stage when curative options are limited [1,2,3].

Alongside this ongoing global challenge, modern healthcare increasingly depends on integrating large and diverse data types—such as Computed Tomography (CT), Low-Dose Computed Tomography (LDCT)/Positron Emission Tomography–Computed Tomography (PET-CT) scans, magnetic resonance image (MRI), histopathology, clinical information, and molecular data—to enable earlier detection and more precise decisions. However, manual interpretation alone is difficult due to the sheer amount and variety of data, leading to issues like reader fatigue, differences between observers, and time constraints, which can result in inconsistent assessments or overlooked findings during screening and diagnosis [3,4]. Artificial Intelligence (AI) and Machine Learning (ML) have therefore been advanced not simply as automation, but as a means to standardize and scale interpretation while surfacing high-dimensional image features that are often imperceptible to humans [2,4].

From early radiomics pipelines to end-to-end deep learning (DL), recent studies demonstrate performance gains for nodule detection, malignancy risk modeling, and segmentation—and, increasingly, for prognosis and treatment-response prediction [2,3,5]. Causey et al. (2018) [6] introduced NoduleX, which fuses Convolutional Neural Network (CNN) features with quantitative imaging (radiomics) and reported Area Under the Curve (AUC) ≈ 0.99 for malignancy discrimination on Lung Image Database Consortium and Image Database Resource Initiative (LIDC-IDRI)—illustrating the discriminative power of learned representations under curated conditions [6]. Content Building beyond imaging alone, contemporary work leverages whole-slide histopathology with graph-attention multiple-instance networks to forecast disease-free survival, signaling a shift toward truly multimodal prognostics that enrich clinical decision-support [7]. Complementing these exemplars, broad syntheses show that DL-based nodule risk models frequently outperform conventional scores and can reduce false-positive callbacks in LDCT workflows; yet they also stress evidence gaps—particularly the scarcity of multi-site external validation and calibration that are prerequisites for routine use [3,8]. In parallel, newer reviews catalogue how CNNs, Recurrent Neural Networks (RNNs), and Generative Adversarial Networks (GAN)-aided pipelines now span detection, segmentation, and classification and increasingly connect to treatment–response modeling and broader decision-support, underscoring a trajectory from “hype” to workflow-aware tools that triage worklists, stabilize reader performance, and integrate heterogeneous data streams [2,3,5]. These advancements align with comparative reviews like Huang et al. (2024) [9] and Kozuka et al. (2020) [10] and application-oriented studies such as Hendrix et al. (2023) [11] and Marcinkiewicz et al. (2024) [12] collectively suggest that artificial intelligence is evolving from a stage of “hype” to a practical decision-support tool—provided that requirements for external validation, calibration, and integration safeguards are adequately addressed.

While several earlier reviews have discussed artificial intelligence and machine learning in thoracic oncology, many remain fragmented, addressing isolated domains such as radiomics, screening, or algorithmic development. Few have connected these elements into a cohesive overview spanning detection, diagnosis, staging, and prognosis within a clinical workflow context. Furthermore, the rapid methodological advances achieved between 2018 and 2025, including deep learning architectures, multimodal data integration, explainable AI, and real-world validation, have significantly reshaped the evidence base. An updated, narrative synthesis is therefore timely to consolidate these developments, identify persistent gaps, and outline strategies for translating AI innovations into routine clinical practice.

This review contributes to the field by consolidating recent AI/ML advances across the lung cancer care continuum, detection, staging, and prognosis, within a clinical workflow context. It addresses pressing needs for early and accurate diagnosis, reduction in inter-reader variability, and integration of multimodal data for personalized treatment planning. By mapping current capabilities, limitations, and future directions, the review provides a practical roadmap for researchers and clinicians to evaluate, validate, and implement AI tools safely and effectively in routine practice.

### 1.1. Background on Lung Cancer Epidemiology and Clinical Challenges

Despite screening gains, late presentation and heterogeneous nodule behavior persist as clinical realities. LDCT programs detect many small or ground-glass-predominant lesions with uncertain natural history; managing these nodules requires consistent measurement (diameter/volumetry), longitudinal comparison with priors, and risk contextualization by age, smoking history, and comorbidities [2,3]. Reader workload is substantial: a single LDCT involves hundreds of slices, and large programs scale to thousands of studies per week, making fatigue and inter-observer variability recurrent challenges [2,13].

Upstream, determining who should be screened remains a significant systems-level challenge. Callender et al. (2023) [1] introduced efficient ensemble machine learning models that simplify eligibility assessment compared to complex tools such as PLCOm2012, representing a practical step toward expanding screening in resource-limited environments. These challenges are particularly relevant to Qatar and the wider MENA region, where lung cancer ranks among the top causes of cancer mortality and screening programs are still developing. Limited access to subspecialty radiologists and uneven diagnostic infrastructure mirror broader constraints observed across many LMIC contexts. Implementing validated AI-assisted imaging and triage tools could therefore enhance early detection, reduce inter-observer variability, and support equitable access to precision oncology in resource-variable healthcare systems.

Downstream, cancer care has become increasingly multimodal: accurate Tumor–Node–Metastasis (TNM) staging often requires combining Computed Tomography (CT), PET-CT, and histopathology data, while risk assessment and treatment selection benefit from incorporating clinicopathologic and, when available, molecular information. Both narrative and systematic reviews like Huang et al. (2024) [9] and Kanan et al. (2024) [14] highlight that manually integrating such diverse data sources is difficult to standardize and scale precisely where AI- and ML-based decision-support systems offer the greatest advantage.

### 1.2. Motivation for AI/ML Applications in Lung Cancer

#### 1.2.1. Throughput and Standardization

DL-based detection and triage can pre-screen or prioritize studies, stabilize reader performance, and reduce oversight, especially for subtle or part-solid nodules [10,11,13]. Computer-aided detection (CAD) and segmentation (e.g., modern 3D U-Nets/ResNets) provide reproducible measurements for Lung-RADS-aligned follow-up and surgical planning, while radiomics/DL hybrids can quantify morphology and texture that are difficult to capture consistently by eye [3,15].

#### 1.2.2. False-Positive (FP) Reduction and Risk Discrimination

LDCT programs face FP friction. Large-scale DL pipelines [16]; later replications in Jacobs et al. (2021) [13] demonstrate that combining detector + malignancy risk modules with priors can lower FP callbacks while maintaining sensitivity. Studies using PET-CT radiomics [12] and hybrid classifiers [11] show that multimodal features sharpen benign–malignant separation, further reducing unnecessary procedures. Reviews like Huang et al. (2024) [9] and Kanan et al. (2024) [14] summarize consistent gains across benchmark datasets, while cautioning that external validation and calibration are critical for net clinical benefit across sites.

#### 1.2.3. Beyond Detection: Prognosis and Response

Prognostic modeling now spans imaging + pathology [7], clinicoradiomic risk models [15], and treatment-response prediction. Such models aim to anticipate survival and recurrence risk, triage adjuvant therapy, and flag high-risk surveillance candidates—linking detection to patient-level decision support.

#### 1.2.4. Data Integration and Real-World Fit

Contemporary reviews such as Huang et al. (2024) [9] and Kanan et al. (2024) [14] and clinical guidance papers like Jensen et al. (2024) [17] and Jeon et al. (2025) [18] converge on a practical agenda: harmonize imaging protocols; adopt prospective, multi-institutional validation; embed AI outputs (probabilities, malignancy scores, calibrated thresholds) into structured reports and navigable dashboards; and monitor impact via quality metrics (FP rate, time-to-diagnosis, stage shift). Feasibility work in registry/Electronic Medical Record (EMR)-augmented cohorts [19,20] suggests that parsimonious models using routinely collected variables can coexist with high-capacity DL by addressing different parts of the pathway (eligibility vs. image triage vs. prognosis).

### 1.3. Scope and Objectives of the Review

This review synthesizes AI/ML advances across the lung-cancer pathway with three objectives. First, we contextualize why AI is needed by outlining the epidemiologic burden and the practical bottlenecks of current clinical practice (screening volume, reader variability, and false-positive management). Second, we survey contemporary AI techniques for imaging-based tasks—pulmonary nodule detection, false-positive reduction and malignancy classification, and segmentation—highlighting representative models and reporting trends drawn from large cohorts and influential reviews (e.g., high-sensitivity CNN detectors; DL+ radiomics hybrids; external-validation gaps and calibration issues). Third, we examine how AI supports risk prediction and prognosis, including radiomics-based risk models, survival modeling, and the integration of multi-modal clinical and imaging data toward precision medicine. Throughout, we prioritize studies that clarify generalizability (multi-center data, external validation), clinical utility (impact on reader performance or downstream decision-making), and interpretability (explainable AI elements), and we frame limitations that must be addressed for safe deployment (harmonization, reproducibility, calibration, and regulatory considerations).

In addition to imaging-centric AI, we map where decision support intersects staging and diagnosis, emphasizing use cases in non-small-cell lung cancer (NSCLC) such as differentiation of subtypes, nodal assessment, and integration with PET-CT and histopathology. Finally, we provide a forward-looking discussion of challenges—data heterogeneity, domain shift, and real-world validation—and propose future directions that can connect high experimental performance to reliable clinical benefit. This scope mirrors the structure of the article: after methodology, we discuss detection and classification (with an emphasis on false-positive reduction), risk prediction and prognosis (radiomics-based and survival models), staging and diagnosis (DL for NSCLC staging; radiomics for malignancy and lymph-node assessment), and conclude with challenges, limitations, and future directions, including interpretability, regulation, reproducibility, and the path toward intelligent, personalized, and real-time clinical integration.

## 2. Materials and Methods

A methodical strategy was used to find, assess, and compile pertinent research examining the uses of AI and ML in lung cancer in order to guarantee methodological rigor and transparency. In order to choose high-quality and clinically relevant publications, the process involved a thorough literature search followed by the application of predetermined inclusion and exclusion criteria. Lastly, to showcase developments in prognostic modeling, diagnosis, and detection, the relevant articles were arranged thematically. Each phase of this process is described in depth in the ensuing subsections.

### 2.1. Literature Search Strategy

To find pertinent papers published between 2018 and 2025, a thorough literature search was carried out across several academic databases, including PubMed, Scopus, IEEE Xplore, and Google Scholar. Combinations of important terms associated with machine learning, artificial intelligence, and lung cancer were used in the search approach. (“lung cancer” OR “non-small cell lung cancer” OR “NSCLC” OR “small cell lung cancer” OR “pulmonary nodule” OR “thoracic oncology”) AND (“artificial intelligence” OR “machine learning” OR “deep learning” OR “radiomics” OR “neural networks”) AND (“imaging” OR “screening” OR “detection” OR “classification” OR “prognosis” OR “staging” OR “survival prediction”).

To guarantee thorough coverage of pertinent material, the search procedure was methodical. Titles and abstracts were checked for relevancy after duplicates were eliminated, and then full-text assessments of potentially eligible publications were conducted. In order to identify new trends and cutting-edge approaches, the reference lists of the included papers and recent reviews were carefully reviewed.

Using well-known frameworks like PROBAST and QUADAS-2, methodological soundness and reporting transparency were qualitatively evaluated during screening, with an emphasis on performance reporting, validation design, and dataset description clarity. There was no official numerical score system used.

The majority of the included papers employed popular public datasets that are crucial to AI-based lung cancer research, including NLST, LUNA16, and LIDC-IDRI. However, these databases frequently lack racial and scanner variety because they are largely made up of Asian or North American cohorts. The quantity and scope of external validations were constrained, and there were not many multi-institutional or real-world datasets accessible. The potential dataset and validation bias introduced by these factors are covered in more detail in upcoming sections.

### 2.2. Summary of Literature Screening and Study Distribution

Database searches were performed throughout PubMed, Scopus, IEEE Xplore, and Google Scholar, yielding 141 entries in total. Following the elimination of 18 duplicate entries, 123 distinct papers were filtered based on their abstract and title; 22 of them were disqualified for failing to satisfy the inclusion requirements. The eligibility of the remaining 101 full-text publications was assessed.

**Figure 1:**
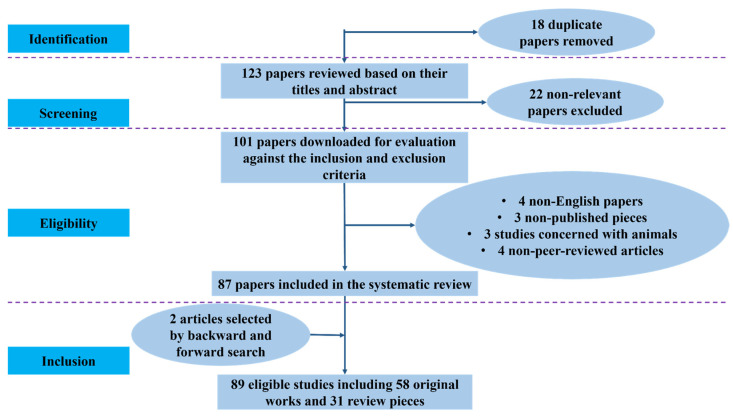
PRISMA flowchart of literature selection.

Four non-English publications, three unpublished works, three animal-based research, and four non-peer-reviewed articles were among the fourteen studies that were eliminated during this phase. In the end, 87 studies were included in the systematic synthesis after meeting all inclusion requirements. Backward and forward reference searching turned up two more papers, bringing the total number of qualifying studies to 89, which included 31 review papers and 58 original research articles. To guarantee openness and repeatability in the identification and selection of studies, the selection procedure, as shown in Figure 1, followed PRISMA-style guidelines.

Detection and Screening (33 studies), Risk Prediction and Prognosis (28 studies), and Staging and Diagnosis (28 studies) are the three areas into which the included studies were thematically arranged to aid in interpretation.

The distribution of records by database, screening stage, and thematic category is compiled in Table 1.

### 2.3. Inclusion/Exclusion Criteria

To guarantee the methodological quality and applicability of the research included in this review, specific inclusion and exclusion criteria were developed.

Criteria for Inclusion:

- Research using AI, ML, or DL methods for the diagnosis, categorization, staging, or prognosis of lung cancer.
- Research using AI-based techniques for image analysis, such as radiomics, feature extraction, segmentation, or combining imaging with molecular or clinical data.
- Original research papers that summarize AI/ML applications in lung cancer, including prospective, retrospective, cross-sectional, or model development investigations, as well as narrative or systematic reviews.
- English-language publications.
- Research that offers quantitative results (like diagnostic accuracy, predictive performance, or survival measures) and well-defined input data (like CT, PET, MRI, histology, or clinical data).
- Publications published from 2018 to 2025 in order to keep up with the latest trends and guarantee their applicability today.

Criteria for Exclusion:

- Research that does not use AI, ML, or DL algorithms for analysis or prediction.
- Studies were conducted on cancers other than lung cancer, such as colorectal, breast, or prostate cancer.
- Abstracts from conferences, letters, editorials, or commentary that do not provide enough quantitative data or methodological information.
- Publications that are not written in English.
- Studies that impede assessment or reproducibility due to inadequate model descriptions, unclear outcome measures, or inadequate data reporting.
- Redundant or overlapping research, unless it offers new information, larger datasets, or a significant shift in analytical viewpoints.

### 2.4. Approach for Organizing Themes

The examined research papers were methodically grouped into topical themes according to their key therapeutic goals and methodological focus in order to guarantee a cogent synthesis of the various uses of AI and machine learning in lung cancer. Each study was assessed for its primary contribution following a first screening and full-text evaluation, regardless of whether it focused on prognostic modeling, diagnostic staging, or early detection. Three broad themes were then identified from the chosen literature:

1. AI in Lung Cancer Detection and Screening: this includes research on deep learning architectures for lung nodule recognition, picture segmentation, and false-positive reduction.
2. AI in Risk Prediction and Prognosis, comprising studies that used survival analysis frameworks, multimodal data integration, and radiomics features to create or test predictive models.
3. Malignancy grading, tumor classification, NSCLC staging, and comparison with traditional radiologists’ evaluations are all covered by AI in Lung Cancer Staging and Diagnosis.

An organized narrative that emphasizes methodological diversity, technological advances, and therapeutic relevance within each subject was made possible by this theme framework. Additionally, in order to address cross-cutting concerns like data heterogeneity, model interpretability, and ethical considerations, the challenges and future directions section was created to incorporate ideas from all three areas. This strategy guarantees a thorough but targeted discussion of how AI and ML are changing the way lung cancer is managed throughout the treatment continuum.

## 3. AI in Lung Cancer Detection and Screening

AI and ML have emerged as transformative tools in lung cancer screening, providing automation, accuracy, and reproducibility in detecting and characterizing pulmonary abnormalities. Traditional LDCT screening remains the clinical gold standard for early detection, yet manual interpretation is time-consuming, subjective, and prone to inter-reader variability. The rapid evolution of AI-driven methods—particularly those employing DL—has considerably improved detection precision and diagnostic efficiency, bridging the gap between radiological expertise and computational intelligence.

The scope of this section encompasses the role of AI in (i) pulmonary nodule detection, (ii) segmentation techniques using deep learning architectures, and (iii) false positive reduction and classification. Collectively, these technological developments signify a paradigm shift toward more consistent and objective lung cancer screening workflows.

We acknowledge that chest X-rays are still frequently used in many healthcare settings and that CAD systems for X-ray imaging have also been thoroughly studied, even though this review focuses on AI applications in LDCT due to its higher sensitivity and its crucial role in contemporary lung cancer screening programs. Although a thorough examination of X-ray-based screening was outside the purview of this LDCT-focused study, we recognize its clinical significance and emphasize it as a crucial complementary path for further research.

### 3.1. Pulmonary Nodule Detection

Pulmonary nodules are small, round opacities often serving as the earliest radiologic indicator of lung cancer. Detecting these nodules accurately on LDCT is critical for early diagnosis and improved patient survival outcomes. AI-based CAD systems now offer substantial improvements in both sensitivity and efficiency compared to conventional manual reviews.

**Figure 2:**
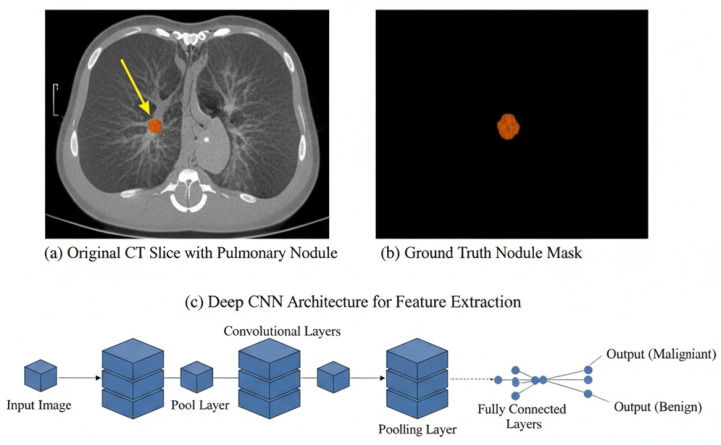
Workflow of deep learning-based pulmonary nodule detection and classification.

Early efforts, such as Causey et al. (2018) [6], introduced NoduleX, a deep learning framework integrating CNN features with quantitative image features (radiomics and texture descriptors). This hybrid model achieved an AUC of 0.99, demonstrating exceptional malignancy prediction accuracy and outperforming radiologist interpretation in consistency and speed. Similarly, Cui et al. (2020) [21] developed a ResNet-50-based algorithm trained on LDCT scans that automatically detected pulmonary nodules with high precision across diverse datasets. Ren et al. (2020) [22] presented the Manifold Regularized Classification-DNN (MRC-DNN) for classifying lung nodules on 3D CT images as benign or malignant, incorporating manifold learning regularization to prevent overfitting in limited datasets. Figure 2 shows a workflow of deep learning-based pulmonary nodule detection and classification. The yellow arrow in the picture (a) is pointing out a pulmonary nodule (red colored), which is a small, abnormal spot or growth seen inside the lung on the CT scan. This nodule is the key focus of the image. The picture (b), labeled “Ground Truth Nodule Mask,” simply shows this same abnormal spot isolated against a black background, which is a method used to precisely highlight and confirm its location and size for analysis.

Large-scale validation studies, including Vachani et al. (2025) [23], reinforced the clinical viability of AI in real-world settings. The REALITY trial, involving 1147 patients across multiple centers in the U.S. and Europe, evaluated an AI/ML algorithm for LDCT-based detection and characterization of pulmonary nodules. Moreover, Jacobs et al. (2021) [13] employed multi-view convolutional networks, which synthesized multiple perspectives of each nodule to lower false-positive rates, enhancing the overall screening reliability. Datasets such as LIDC-IDRI, LUNA16, and NLST remain instrumental in benchmarking AI algorithms, enabling cross-validation and ensuring methodological rigor. These studies collectively demonstrate strong performance in curated datasets, with CNN-based models achieving high sensitivity and specificity. Strengths include robust feature extraction and integration of radiomics for improved malignancy discrimination. However, most models were trained on single-center or competition datasets, limiting generalizability. External validation remains rare, and interpretability is minimal, which may hinder clinical trust. Additionally, computational complexity and reliance on large annotated datasets pose practical challenges for deployment in resource-limited settings. Recent AI-based approaches for pulmonary nodule detection with performance metrics are shown in Table 2.

Across these studies, several strengths stand out. Many CNN-based detection models demonstrate high diagnostic accuracy on curated datasets, and some multimodal approaches show meaningful gains when imaging is combined with clinical or pathological information. A few recent multi-centre evaluations also provide early evidence that these systems can perform well beyond a single institution. At the same time, the literature shows important limitations. A large proportion of models are trained on single-centre data, which narrows their generalisability, and variability in imaging protocols often complicates comparisons across studies. In many cases, interpretability remains limited and prospective validation is still lacking, which makes it difficult to fully judge how these methods would behave in routine clinical settings.

### 3.2. Segmentation Techniques Using DL Architectures

Segmentation serves as the cornerstone of quantitative lung imaging, enabling precise localization and volumetric measurement of nodules or tumors. Effective segmentation supports downstream analyses such as malignancy classification, growth tracking, and treatment monitoring. Recent DL models have markedly enhanced segmentation accuracy, overcoming challenges of image heterogeneity, low contrast, and anatomical overlap. The U-Net architecture and its variants 3D U-Net, V-Net, and Res U-Net have proven particularly effective. For instance, Yu et al. (2021) [24] proposed a two-part CAD system integrating a 3D Res U-Net for segmentation and a 3D ResNet-50 for classification, achieving a Dice coefficient exceeding 0.8 for nodules larger than 10 mm. Hybrid CNN–transformer architectures incorporating attention mechanisms have further refined boundary detection and minimized segmentation errors due to noise or artifacts.

Strengths of these approaches include high segmentation accuracy, improved volumetric consistency, and adaptability to complex lung structures. Attention-based and multi-view models demonstrate superior performance in delineating small or irregular nodules compared to classical architectures. However, weaknesses persist: most studies rely on curated datasets with limited diversity, raising concerns about generalizability. Transformer-based models, while powerful, demand large datasets and significant computational resources, which may hinder clinical scalability. Furthermore, interpretability remains limited, and federated learning frameworks though promising for privacy-preserving multi-institutional training are still in early stages with technical and regulatory barriers.

### 3.3. False Positive Reduction and Classification

Reducing false positives (FPs) has become a defining performance criterion for contemporary AI-driven systems that detect lung nodules, given that excessive false alarms undermine radiologist confidence, precipitate avoidable downstream investigations, and impede the broader clinical adoption of computer-aided screening tools [25]. Modern algorithms therefore employ multi-stage detection frameworks in which an initial high-sensitivity candidate generator—typically a 3D convolutional or region-proposal-based detector—identifies all potential nodules, followed by a specialized FP-reduction module designed to eliminate structures commonly misclassified as nodules, including vascular bifurcations, airway walls, fissural intersections, pleural thickenings, and motion- or noise-related artifacts. Across the datasets summarized in Table 2, a clear methodological gradient is apparent: architectures that explicitly incorporate volumetric contextual reasoning, attention mechanisms, and multimodal feature integration consistently demonstrate the most effective FP suppression [26]. On widely used public LDCT datasets such as LUNA16 and LIDC-IDRI, attention-enhanced 3D models—including Mask R-CNN derivatives, 3D ECA-ResNet, and PiaNet—frequently achieve high sensitivity while maintaining false positive rates at or below approximately one FP per scan [27]. Their performance advantage stems from their capacity to model interslice continuity, encode peri-nodular anatomical context, and capture fine-grained textural and morphological characteristics that differentiate true nodules from benign mimics [28]. In contrast, earlier generations of models—including classical ResNet-based classifiers, shallow 3D CNNs, and manifold-regularized deep networks—although often demonstrating strong overall accuracy and respectable specificity, exhibit substantially higher or more variably reported FP rates when evaluated on LIDC-IDRI and multi-institutional cohorts [29]. This variability reflects their limited ability to represent complex thoracic anatomy, insufficient modeling of adjacency to vascular or pleural structures, and reduced robustness to variations in reconstruction parameters. Hybrid pipelines that fuse handcrafted radiomic descriptors (e.g., shape, margin, heterogeneity, texture) with deep convolutional embeddings further enhance FP reduction, especially for diagnostically challenging categories such as ground-glass opacities, part-solid nodules, and juxtavascular lesions, where single-modality representations may be insufficient [30]. Nonetheless, the favorable FP-reduction results observed in curated public datasets do not automatically translate to heterogeneous real-world LDCT environments. Multi-center cohorts introduce pronounced variability in slice thickness, scanner models, reconstruction kernels, patient demographics, disease prevalence, and annotation standards—all of which exacerbate domain shift and can elevate FP rates [31]. Consequently, although current evidence robustly supports the superiority of 3D attention-based and multimodal FP-reduction architectures, rigorous external validation, domain-adaptation strategies, and prospective multi-center trials remain essential to determine their reliability, reproducibility, and clinical utility in routine lung cancer screening practice [32].

**Table 2:** Recent AI-based approaches for pulmonary nodule detection and performance metrics.

Reference	Algorithm	Source of Data	No. of Cases	Type of Validation	Main Finding	Quality Index Value
Cai et al. (2025) [5]	Mask R-CNN with ResNet50 architecture	Data from LUNA16 dataset	888 patients from the LUNA16 dataset	800 patients from an independent dataset from the Ali TianChi challenge	Using mask R-CNN and the ray-casting volume rendering algorithm can assist radiologists in diagnosing pulmonary nodules more accurately.	Mask R-CNN of weighted loss reaches sensitivities of 88.1% and 88.7% at 1 and 4 false positives per scan
Ren et al. (2020) [22]	MRC-DNN	Data from LIDC-IDRI dataset	883 patients from the LIDC-IDRI dataset	98 patients from the LIDC-IDRI dataset	MRC-DNN facilitates an accurate manifold learning approach for lung nodule classification based on 3D CT images	The classification accuracy on testing data is 0.90 with sensitivity of 0.81 and specificity of 0.95
Cui et al. (2020) [21]	ResNet	Lung cancer screening data from three hospitals in China	39,014 chest LDCT screening cases	Validation set (600 cases). External validation: the LUNA public database (888 studies)	The DL model was highly consistent with expert radiologists in terms of lung nodule identification	The AUC achieved 0.90 in the LUNA dataset
Yu et al. (2021) [24]	3D Res U-Net	LIDC-IDRI	1074 CT subcases from LIDC-IDRI	174 CT data from 1074044 CT subcases	3D Res U-Net can identify small nodules more effectively and improve its segmentation accuracy for large nodules	The accuracy of 3D ResNet50 is 87.3% and the AUC is 0.907
Yuan et al. (2024) [26]	3D ECA-ResNet	LUNA16/LIDC-IDRI	1080 scans/888 scans	Comparison with state-of-the-art methods.	Multi-modal feature fusion of structured data and unstructured data is performed to classify nodules	Accuracy (94.89%), sensitivity (94.91%), and F1-score (94.65%) and lowest false positive rate (5.55%).
Liu et al. (2023) [27]	PiaNet	LIDC-IDRI	302 CT scans from LIDC-IDRI	52 CT scans from LIDC-IDRI	Pi-aNet is capable of more accurately detecting GGO nodules with diverse characteristics.	A sensitivity of 93.6% with only one false positive per scan

## 4. AI in Risk Prediction and Prognosis

Artificial intelligence has become central to estimating malignancy risk and predicting outcomes in lung cancer. Across contemporary evidence, three complementary themes recur. First, radiomics pipelines convert chest CT and PET/CT scans into quantitative features that characterize nodule shape, intensity, and texture, then learn patterns linked to malignancy risk and biological behavior. Second, time-to-event modeling uses imaging, pathology, and clinical data to predict survival endpoints such as overall survival, progression-free survival, and cancer-specific outcomes. Third, multimodal integration combines images with tabular clinical variables, laboratory markers, histopathology, and genomic signals to improve discrimination, calibration, and generalizability in real-world settings. These themes are consistent across reviews and original studies in the literature and collectively point toward individualized risk estimation and prognosis that move beyond stage-only rules or single-source baselines [15,32].

Hand-crafted radiomics with classical machine learning and deep learning systems trained end-to-end both report gains over traditional assessment when pipelines are designed carefully and inputs are harmonized. Several works note that modest clinical fusion, even with a small set of covariates such as age, smoking, and stage, gives a consistent boost over imaging alone. These themes repeat in malignancy-risk classification, in survival modeling, and in surrogate outcome settings around the peri-operative pathway [33,34]. The most robust studies also describe external validation or multi-institutional training, which reduces sensitivity to specific scanners or protocols and makes reported performance more believable for translation. Table 3 summarizes common data modalities used for lung-cancer risk prediction and prognosis, typical AI/ML methods applied to each, and their main strengths and limitations.

### 4.1. Radiomics-Based Risk Models

Radiomics studies in the literature generally follow a reproducible pipeline: image standardization, segmentation, feature extraction, selection, and supervised modeling. Features often include first-order statistics, shape descriptors, gray-level co-occurrence and run-length textures, and filtered transforms that amplify edge or frequency information. Reviews emphasize that feature stability and preprocessing matter because downstream discrimination depends on how consistently images are acquired and processed. When features are stable, even simple models such as penalized logistic regression or SVM can compete well [15,32].

Several papers extract hundreds to more than a thousand radiomic variables per nodule, then apply dimensionality reduction with methods like Least Absolute Shrinkage and Selection Operator (LASSO) or embedded selection in tree-based learners. That pipeline appears in studies that start with large PyRadiomics panels and end with compact signatures that classify malignancy or reflect biology such as PD-L1 status. Hybrid modeling is common. One strand builds a feature signature with LASSO and then trains a logistic or XGBoost classifier. Another learns deep features with a CNN and combines them with a hand-crafted signature. Both report useful gains, and they tend to improve further when a small clinical vector is concatenated at the model stage [33,35].

There is consistent evidence that radiomics can outperform rule-based triage such as Lung-RADS when models are tailored to nodule type and attenuation. Reviews that summarize head-to-head comparisons report higher AUCs for radiomics-based approaches, especially in indeterminate nodules where texture and shape cues are informative beyond size alone. The evidence also supports longitudinal designs. Serial CT features capture change in density, margin, and internal heterogeneity, which relate to growth and aggressiveness. Papers that include peri-interval scans like de Margerie-Mellon & Chassagnon (2023) [32] and Gandhi et al. (2023) [34] often describe improved discrimination and more stable decision thresholds compared with single-timepoint analysis.

**Figure 3:**
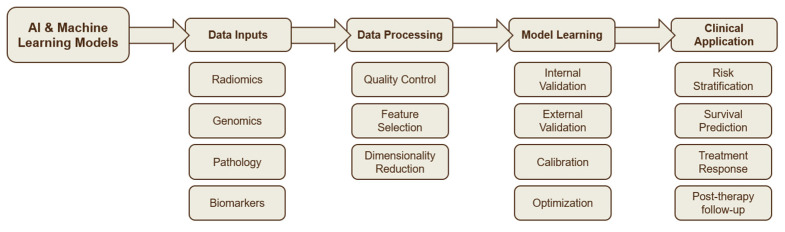
AI-driven framework integrating multi-modal data for risk prediction and prognostic modeling in lung cancer.

Another recurring point is complementarity between hand-crafted and deep radiomics. CNN features trained for classification learn hierarchical morphology that is different from the hand-crafted texture family. When fused, the combined representation can be more robust than either alone. Studies show this pattern for malignancy risk, recurrence prediction, and response modeling. Gains can also appear when radiomics is layered on top of TNM or histology, which reflects the idea that quantitative imaging adds information that is not captured by stage or subtype labels alone [41,42]. Multimodal versions of these models are widely favored, with multiple papers noting that simple clinical fusion improves both discrimination and calibration compared with image-only baselines [43,44]. Figure 3 presents an integrated framework illustrating how artificial intelligence unites imaging, clinical, and molecular data streams to support risk prediction and prognostic modeling in lung cancer.

### 4.2. Survival Prediction Models

Survival modeling spans classical statistics and deep learning. Cox proportional hazards and its penalized variants are used across many studies. Random survival forests, gradient-boosted survival, and DeepSurv appear often, especially when feature spaces are high-dimensional. The inputs range from pure radiomics to deep features, to mixed sets that include stage, histology, and treatment descriptors. Many papers show that once a well-selected imaging signature is combined with a concise clinical vector, risk stratification improves beyond clinical-only models and can be sustained across internal splits or external cohorts [37,40,45].

A clear theme is the benefit of longitudinal information. Models that include repeated CT or peri-treatment imaging achieve stronger discrimination than single-timepoint approaches. This is plausible because temporal change reflects therapy effect and evolving tumor biology, which influence outcomes. Reviews that summarize time-series or follow-up imaging consistently report better risk separation and more clinically meaningful decision thresholds. Longitudinal data is also used in peri-operative paths where short-term radiologic response and early recurrence are central outcomes [46,47,48,49].

Survival modeling beyond radiology is also documented. Graph-based embeddings transform gene panels into patient-level vectors that capture network structure, then train survival learners on these representations. This line appears in immuno-oncology contexts and in general NSCLC cohorts. Studies that compare deep survival models with regularized Cox often find that deep models do well when data volume supports them, but the gap narrows when careful feature selection and regularization are applied in classical frameworks. This is encouraging for translation because it suggests that stable performance is achievable without excessive complexity if features are engineered and selected well [37,39].

Endpoint choice varies and is tied to clinical context. Overall survival and progression-free survival dominate, but there is growing interest in event-free survival, disease-free survival, major pathologic response, and pathologic complete response as surrogate endpoints in neoadjuvant and peri-operative studies. These choices are logical for treatment evaluation where long follow up is impractical. Authors stress validation and calibration checks for these surrogates, so models trained on one program do not overstate performance when moved to another [18,38]. When survival models are applied without proper external validation and calibration, their predictions may not match the patients they are being used for. This can lead to risk estimates that are higher or lower than they should be, which affects how clinicians judge urgency, plan treatment, and decide who needs closer follow-up. Even small differences between predicted and actual outcomes can influence decisions in ways that may not serve patients well. To ensure these models are truly safe in practice, they need to be tested and calibrated on independent patient groups before being utilized in real clinical settings. Review papers reinforce the need for clear reporting of validation design and transparent performance summaries, especially when models are compared across sites or when imaging protocols differ [14,50,51].

### 4.3. Integration of AI with Clinical and Imaging Data

Integration is one of the most consistent findings across the evidence base. Many pipelines combine CT or PET/CT features with demographics, smoking history, stage, histology, and simple laboratory markers. This light multimodal fusion is often enough to improve both discrimination and calibration compared with single-source models. It also reduces sensitivity to specific scanners because clinical variables carry signals that are independent of image acquisition [52,53]. The same reasoning appears in studies that enlarge the fusion set to include pathology or selected genomics. When these inputs are added in a structured way, performance gains are observed without loss of interpretability, especially if the clinical side is limited to a concise set of high-signal covariates [7,54].

Several integration examples in the literature highlight two distinct design choices. The first is early feature fusion, where image features and clinical variables are concatenated before the learner. The second is late fusion, where separate sub-models are trained and their risk scores are combined by a meta-learner or a calibrated rule. Early fusion is simple and works well when data is clean and missingness is limited. Late fusion is useful when modalities are imbalanced or missing at different rates, which is common in retrospective cohorts. Both designs are represented and both show advantages in different settings. In either case, small sets of clinical covariates aligned with known prognostic factors usually deliver most of the benefit [55,56,57].

A number of papers go beyond feature fusion and describe integration at the workflow level. Outputs are surfaced to radiologists or tumor boards as calibrated risk scores or risk strata, sometimes with simple explanations such as top contributing features or prototypical image patches. This is especially clear in studies that are trained on multi-institutional data. These groups emphasize robustness to domain shift by integrating the model into the existing reading and decision pathway rather than replacing it. The result is a system that does not rely on narrow input distribution and that offers value even when images come from different vendors or when tabular variables are incomplete [23,58,59].

Integration is not limited to clinical variables. Several papers discuss adding pathology or weakly supervised histology signals to imaging models. Others bring in limited genomic panels or knowledge-guided graph embeddings that summarize relationships among mutations, pathways, and therapies. These strategies aim to connect morphology with biology in a compact way that can be learned from modest sample sizes [60,61]. The literature documents that graph-based representations can feed standard survival learners or be combined with imaging features in a two-branch network. Studies that use these designs report improved stratification, especially for treatment response or immuno-oncology subcohorts, with an emphasis on keeping the clinical branch concise to reduce overfitting risk [62,63,64].

There are also examples where integration favors practicality over breadth. Some studies use only tabular clinical data when imaging is unavailable and still produce useful triage risk. These models are not as strong as multimodal systems when matched head-to-head, but they are quick to deploy and easy to maintain [65,66]. Conversely, imaging-only models can serve as a baseline when tabular data are fragmented or when pathology and genomics are missing. Many groups argue for a staged approach in which a simple clinical model triages, an imaging model refines the estimate, and a multimodal system is used when all inputs are present. This staged design respects real-world data quality without sacrificing performance when richer inputs are available [19,67].

The literature also includes integration across institutions and scanners. Multi-institutional training or evaluation is a form of integration that blends data from different centers by design. This broadens the image and clinical distributions and makes final models less brittle. Studies that report this design often note that discrimination holds up better on external cohorts and that calibration shifts are smaller and easier to correct. Several of these works describe harmonization steps such as intensity standardization or scanner-aware augmentation [26,36,68]. Others prefer to keep preprocessing minimal and rely on clinical fusion and diverse sampling to achieve robustness. Both strands are represented and both improve transportability compared with single-center training [23,58,69].

Finally, even when deep models are used, the multimodal block can produce outputs that are easy to communicate, such as a binary risk tier or a calibrated probability with a brief rationale. Imaging contributes structure and texture cues that clinicians recognize, while clinical covariates connect the prediction to familiar risk factors. When these elements are combined, model outputs can be discussed in the same language used for stage and histology, which lowers the barrier to adoption. The evidence shows that this is achievable with relatively simple architectures and does not require exotic modeling choices. The consistent message is that small, well-chosen multimodal inputs make a reliable difference in both risk estimation and prognosis [70,71]. Table 4 summarizes representative AI approaches for lung-cancer risk estimation and prognostic modeling, including their typical inputs, modeling designs, and reported performance advantages.

Despite encouraging progress in multimodal fusion, several barriers continue to limit real-world implementation. Data governance remains a central challenge: institutional privacy regulations, fragmented data ownership, and differing consent frameworks often prevent multi-institutional data sharing essential for robust external validation. Missing variables, particularly incomplete clinical, molecular, or follow-up data, introduce bias and weaken model calibration when transferred to new populations. Moreover, model drift poses an emerging concern, as shifts in imaging protocols, scanner hardware, and patient demographics over time can gradually degrade performance. Without continuous monitoring and periodic retraining, AI systems may deliver inconsistent outputs in evolving clinical environments. Addressing these issues through standardized data pipelines, federated learning, and post-deployment auditing will be crucial to ensure that AI integration in lung cancer care remains reliable and equitable.

## 5. AI in Lung Cancer Staging and Diagnosis

Accurate TNM staging and timely diagnosis remain central to management of NSCLC. Radiologists rely on CT, PET/CT and histopathology, but these processes are labour-intensive and susceptible to inter-observer variability [9,16,20]. AI and ML aim to standardise measurements, automate repetitive tasks and integrate multi-modal information to support clinical decision-making [15,74,75].

AI-assisted imaging does not replace bronchoscopy, biopsy, or molecular testing, but it increasingly supports the pathway that leads to these procedures. Imaging-based algorithms can help identify lesions that warrant prioritised biopsy, estimate the likelihood of malignancy, and highlight features associated with nodal involvement or aggressive behaviour. When combined with pathology and genomic information, multimodal AI models can contribute to decisions regarding surgery, adjuvant therapy, and immunotherapy, especially in settings where risk stratification influences treatment thresholds. In clinical practice, these outputs are most valuable when they complement tissue diagnosis and molecular profiling during multidisciplinary discussions, helping clinicians organise investigations, anticipate management needs, and make more consistent treatment decisions.

### 5.1. Deep Learning Models for NSCLC Staging

DL systems commonly begin with CNNs such as U-Net or Mask R-CNN to segment primary tumours and regional lymph nodes on CT or PET/CT, providing volumetric and morphologic inputs that support T- and N-stage estimation [76,77]. Evidence across recent reviews indicates that pairing accurate segmentation with representation learning improves reproducibility relative to purely visual assessment and helps standardise reporting across institutions [76,78,79]. Clinical narrative reviews further describe pipelines that quantify local invasion patterns and nodal burden in ways that align with contemporary staging practice [34,41].

A complementary set of studies address “staging-adjacent” endpoints that inform stage grouping or treatment planning, such as distinguishing pre-invasive from invasive adenocarcinoma or grading tumour aggressiveness [4,5,43]. Deep-radiomics and classification frameworks trained on annotated cohorts report strong discrimination of invasive disease and histologic subtypes, providing structured inputs that can map onto TNM-relevant decisions in multi-disciplinary care [55,72]. Additional work shows that DL features combined with clinical variables can stratify risk trajectories associated with stage burden and outcomes [80,81]. Risk-prediction studies similarly argue for integrated imaging-plus-clinical modelling to drive stage-aligned decisions and follow-up strategies [43,82,83]. Overall, DL offers standardised lesion delineation and the ability to surface subtle spatial patterns, thereby improving the consistency of inputs that underpin TNM classification, while most reports call for prospective validation and workflow integration to confirm benefits in routine practice [14,84]. Table 5 shows AI domains relevant to NSCLC staging and diagnosis, showing how radiomics, pathomics, genomics, and immunomics contribute to tumor segmentation, histologic classification, and risk prediction.

Beyond algorithmic accuracy, the translational potential of these AI tools lies in their ability to enhance clinical decision support. Integrated within multidisciplinary tumor boards, AI-derived segmentation maps or risk scores can help radiologists, oncologists, and pathologists reach more consistent staging and treatment decisions. Embedding predictive outputs directly into structured radiology reports or electronic medical records could standardize communication of tumor burden, lymph-node involvement, and progression risk. Such systems may also facilitate triage, prioritizing complex or ambiguous cases for expert review, thereby improving workflow efficiency and diagnostic equity. However, real-world deployment will depend on interoperability with existing hospital information systems, clinician training, and clear responsibility frameworks to ensure that AI acts as an assistive, not autonomous, decision partner.

### 5.2. Radiomics for Tumour Malignancy and Lymph-Node Assessment

Radiomics converts CT or PET/CT images into quantitative descriptors—intensity, texture and shape features—followed by ML classifiers or survival models for clinical prediction [14,15]. Multiple studies report accurate discrimination of benign versus malignant nodules and stratification of invasive potential, complementing radiologist assessment in screening and diagnostic pathways [6,49,86]. Extensions across larger or multi-centre cohorts demonstrate robust malignancy scoring and histology-aware characterisation that generalise beyond single-site datasets [54,59]. Expanded pipelines incorporating clinical variables and handcrafted or deep-radiomics features further improve phenotype classification and prognostic modelling [2,76].

Recent contributions emphasise fusion of deep-radiomics with multi-modal inputs and domain adaptation for cross-site generalisation, supporting malignancy assessment and nodal inference in heterogeneous populations [3,26]. Emerging frameworks report improved discrimination after integrating learned features with classical radiomics, suggesting a path toward robust clinical deployment across imaging protocols [5,75]. Survey and methods papers also describe how deep-radiomics can be leveraged for lymph-node risk estimation or as a surrogate outcome that correlates with nodal involvement in comprehensive pipelines [4,55]. Studies on peri-tumoral and multi-structure modelling, as well as outcome-focused radiomics, show that quantitative signatures capture biology relevant to staging decisions and downstream management [72,87]. Complementary work highlights multi-structure and prognostic modelling as an on-ramp for staged care pathways, including mortality risk and treatment selection [32,34]. Risk and follow-up frameworks that incorporate radiomics alongside clinical variables further support stage-aligned management decisions [12,30].

### 5.3. Comparison of AI Models with Traditional Radiologist Assessment

Head-to-head evaluations examine whether AI matches or exceeds expert readers while maintaining acceptable false-positive burdens and interpretability [10,11]. In screening cohorts, top systems trained on large-scale competitions and institutional datasets perform on par with groups of radiologists for cancer risk estimation, with performance varying by lesion subtype and operating point [13]. Broad syntheses consistently find that AI can equal or outperform radiologists for specific tasks—including detection, malignancy scoring and risk prediction—while reader-in-the-loop configurations stabilise false positives and improve acceptance in clinical practice [9,14]. Additional comparative studies extend these findings to triage roles, second-reader assistance and histology-aware modelling in diverse settings, underscoring that the greatest gains arise when AI complements rather than replaces human expertise [40,68].

**Figure 4:**
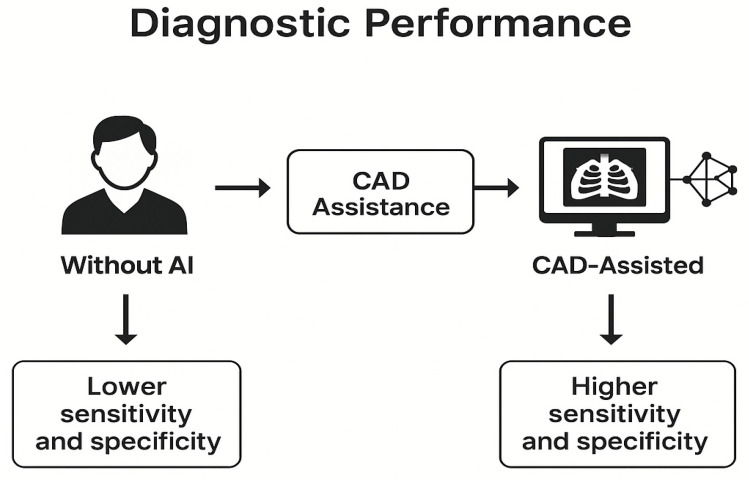
Enhancement of NSCLC with CAD.

The literature indicates that AI—particularly deep learning and radiomics techniques—has the potential to augment lung-cancer staging and diagnosis. Only a handful of papers describe detailed algorithms for automated TNM staging, and only a few radiomics studies report dedicated methods for lymph-node assessment. Most current work either demonstrates proof-of-concept classifiers or provides qualitative descriptions, and robust external validation is rare. Comparative studies consistently show that AI models can match or exceed radiologists in sensitivity, particularly for small or subtle lesions, but concerns persist about false positives, generalisability and interpretability [85,88]. Advancing these technologies into routine care will require larger, standardised datasets, integration of imaging with clinical and molecular information, and transparent algorithms that earn clinician trust. When these challenges are addressed, AI is poised to become a valuable partner to radiologists, enhancing both the accuracy and efficiency of lung-cancer management. Figure 4 shows a simplified schematic illustrating how CAD enhances diagnostic performance in NSCLC by improving radiologist sensitivity and specificity compared to manual image interpretation.

## 6. Challenges, Limitations, and Future Directions

**Figure 5:**
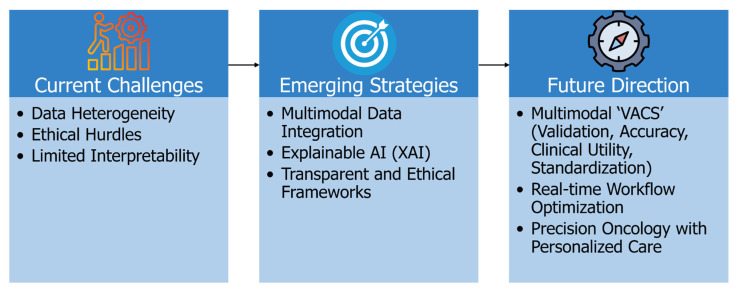
AI in lung cancer care: From proof-of-concept to clinical application.

The incorporation of artificial intelligence into lung cancer care offers transformative potential; however, its clinical adoption remains hindered by enduring challenges. This section consolidates key limitations highlighted in recent studies—such as data heterogeneity, limited model interpretability, and regulatory complexities—while discussing emerging strategies designed to address these obstacles. By exploring future directions including multi-modal integration, personalized treatment planning, and real-time workflow optimization, we propose a roadmap to guide AI’s progression from proof-of-concept to clinical application. Figure 5 visually represents the journey of AI in Lung Cancer Care: From Proof-of-Concept to Clinical Application, specifically highlighting the Challenges, Limitations, and Future Directions.

### 6.1. Addressing Data Heterogeneity for Better Generalizability

AI models developed for lung cancer care frequently exhibit limited generalizability across diverse clinical settings due to underlying data heterogeneity. Prior studies, including those by Hendrix et al. (2023) [11] and Jacobs et al. (2021) [13], demonstrate that variations in CT slice thickness, imaging protocols, and scanner types can substantially influence model performance. For example, Hendrix et al. (2023) [11] reported that the AI model achieved 90.9% sensitivity internally versus 92.4% externally at 1 FP/scan, but thicker 3 mm slices in the external site contributed to higher false negatives for specific nodule types, such as juxtapleural (nA = 17, nB = 11) and juxtavascular nodules (nA = 5, nB = 2). Similarly, Jacobs et al. (2021) [13] found that top-performing algorithms reached AUCs of 0.877–0.902 on an external dataset compared with radiologists’ average of 0.917, with one model performing significantly worse (*p* = 0.02), highlighting variability in external generalization. Marcinkiewicz et al. (2024) [12] reported variability in reporting of incidental findings across sites, indicating that differences in data acquisition and annotation may influence AI performance. Reliance of Kesiku & Garcia-Zapirain (2025) [89] on a single-center dataset further highlights risks of linguistic and institutional bias. Moreover, Huang et al. (2024) [9] and M S et al. (2025) [83] noted the absence of standardized datasets and demographic diversity, constraining external validity. Collectively, these findings emphasize the importance of developing multi-institutional, multilingual, and multimodal datasets to promote robust generalization and equitable AI deployment in lung cancer care.

### 6.2. Enhancing Model Interpretability and Clinical Trust

Interpretability remains a cornerstone for the clinical adoption of AI systems in lung cancer care. Hendrix et al. (2023) [11] and Jacobs et al. (2021) [13] noted that the absence of visual rationales or nodule-level explanations limited radiologist engagement with their models. Conversely, Huang et al. (2024) [9] and Kesiku & Garcia-Zapirain (2025) [89] underscored the value of explainable AI approaches—such as SHAP, Grad-CAM, and attention mechanisms—to elucidate model reasoning. However, these tools may not always provide complete or clinically intuitive explanations, and their usefulness can vary depending on the specific task or model architecture. Explainability remains a critical challenge. Techniques such as SHAP and Grad-CAM are commonly applied to interpret deep learning predictions. SHAP provides feature-level importance scores that reveal which radiomic or image-derived features contribute most to false positive (FP) or true positive classification. For example, margin irregularity or subtle peri-nodular texture may disproportionately drive FP assignments in part-solid or ground-glass nodules, guiding targeted refinement of FP-reduction modules and radiologist review. Grad-CAM generates spatial heatmaps highlighting regions influencing the model’s decision; however, heatmaps can be ambiguous for small, low-contrast, or juxtavascular nodules, sometimes emphasizing vessels or pleura rather than the nodule itself (Marcinkiewicz et al. (2024) [12]; Huang et al. (2024) [9]). SHAP can also be computationally intensive and may oversimplify feature interactions in high-dimensional spaces, potentially masking clinically relevant subtleties. Taken together, these tools should complement expert review rather than replace it, particularly when FP-reduction decisions involve nuanced or context-dependent cases. M S et al. (2025) [83] implemented LIME to generate feature-level insights, while Marcinkiewicz et al. (2024) [12] employed SHAP-based structural attributions to facilitate radiologist interpretation. Furthermore, Jensen et al. (2024) [17] highlighted the role of genomic profiling in enhancing diagnostic confidence when differentiating second primary lung cancers from recurrence. Embedding transparency within model architecture is therefore pivotal for building clinician trust and promoting collaborative decision-making.

These ethical and regulatory considerations are closely linked to practical integration challenges. Weak data governance, incomplete clinical variables, and unmonitored model drift not only reduce reproducibility but also raise accountability and fairness concerns. Implementing continuous post-deployment monitoring, federated data infrastructures, and transparent audit trails can help align ethical compliance with sustained model performance in real-world oncology settings.

### 6.3. Navigating Ethical, Regulatory, and Reproducibility Challenges

Ethical and regulatory frameworks are fundamental to the successful translation of AI models into clinical practice. As highlighted by Huang et al. (2024) [9] and Jacobs et al. (2021) [13], regulatory approval necessitates prospective validation and adherence to evolving standards. Ethical issues—including data privacy, algorithmic bias, and accountability for diagnostic errors—were emphasized by Hendrix et al. (2023) [11] and Marcinkiewicz et al. (2024) [12]. Kozuka et al. (2020) [10] and M S et al. (2025) [83] further identified limited reproducibility as a persistent challenge, citing the lack of publicly accessible code and standardized analytical pipelines. Kesiku & Garcia-Zapirain (2025) [89] underscored the importance of pilot testing and clinician engagement for real-world implementation. Collectively, these findings highlight that transparent reporting, open-source development, and robust ethical oversight are essential to ensuring the safe and equitable adoption of AI in lung cancer care.

### 6.4. Toward Intelligent, Personalized, and Real-Time Clinical Integration

The future of AI in lung cancer care depends on its capacity to provide intelligent, personalized, and real-time support throughout the clinical continuum. Huang et al. (2024) [9] and Jensen et al. (2024) [17] envision multi-modal integration combining imaging, genomic, and clinical data—to improve diagnostic accuracy and disease characterization. Predictive modeling for survival, recurrence, and therapeutic response is gaining momentum, enabling individualized treatment strategies informed by molecular and clinical profiles [18,83]. Real-time clinical deployment is also becoming increasingly achievable, as demonstrated by Hendrix et al. (2023) [11] and Marcinkiewicz et al. (2024) [12], who implemented rapid scan processing and automated visualization overlays. Collectively, these advancements mark a paradigm shift toward precision oncology, where AI complements clinical expertise to deliver timely, patient-centered care.

AI is transforming lung cancer care through improved diagnosis, prognosis, and personalized treatment. Yet, challenges in data quality, interpretability, and regulation persist. Advances in multi-modal integration and explainable, real-time systems are paving the way toward precision oncology and patient-centered care.

Successful clinical integration of AI requires more than algorithmic excellence, it depends on principles of implementation science. Adopting AI tools in lung-cancer care necessitates structured clinician training, workflow adaptation, and digital infrastructure capable of supporting continuous learning systems. Radiologists, oncologists, and data scientists must collaborate to define realistic use-cases, performance monitoring metrics, and feedback mechanisms to ensure sustained model performance. Organizational readiness, encompassing leadership engagement, interoperability of hospital information systems, and staff digital literacy, strongly influences long-term success. Embedding these human and systemic considerations early in AI deployment can bridge the persistent gap between technical feasibility and routine clinical adoption.

## 7. Conclusions

By improving the precision, speed, and repeatability of detection, diagnosis, and prognostic assessment, artificial intelligence and machine learning are revolutionizing lung cancer research and clinical practice. This paper demonstrates how radiomics-driven predictive tools and DL-based imaging models have developed to detect subtle illness patterns that are invisible to the human eye. Better result prediction and more tailored treatment choices have been made possible by combining AI-derived insights with clinical and genetic data. However, resolving enduring issues with data standards, model transparency, and regulatory approval is necessary to fully realize AI’s therapeutic potential. The creation of explainable AI frameworks, extensive multicenter validation, and smooth interaction with current clinical workflows should be the main goals of future research. AI has the potential to transform from an auxiliary tool to a reliable collaborator in precision lung cancer treatment by coordinating algorithmic innovation with ethical and therapeutic criteria. In conclusion, a paradigm change toward more intelligent, data-driven, and patient-centered oncology is represented by the combination of AI, ML, and contemporary medical imaging. This will help to bring the goal of individualized lung cancer management closer to clinical reality.

To accelerate this translation, several concrete steps are required. First, prioritize external, multi-institutional validation to confirm model robustness across scanners, populations, and clinical workflows. Second, establish regional and international AI consortia, for example, within the MENA region, to promote standardized datasets, data governance frameworks, and shared validation protocols. Third, align AI development with national and institutional policy frameworks for digital health, ensuring compliance with ethical, regulatory, and privacy standards. Finally, invest in clinician education and infrastructure to support continuous model monitoring and responsible deployment. Implementing these strategies will transform AI from an experimental tool into an integrated, trustworthy component of precision lung-cancer care.

## Figures and Tables

**Table 1:** Summary of identified, screened, and included articles.

Database	Records Identified	Duplicates Removed	Screened (Title/Abstract)	Excluded	Full-Text Assessed	Included in Review	Detection & Screening	Risk Prediction & Prognosis	Staging & Diagnosis
PubMed	55	11	44	4	40	33	12	10	11
Scopus	40	2	38	10	28	24	8	8	8
IEEE Xplore	26	2	24	5	19	17	7	5	5
Google Scholar	20	3	17	3	14	13	6	5	2
Total	141	18	123	22	101	87 (+2 = 89)	33	28	28

**Table 3:** Modalities and representative AI/ML approaches used for lung-cancer risk prediction and prognosis with strengths and limitations.

Reference	Data Modality	Example Inputs	Common AI/ML Approaches	Strengths	Key Limitations
[15,32,35]	CT imaging (radiomics)	Nodule texture, shape, attenuation/density	CNNs (incl. 3D), ResNet/U-Net (segmentation), XGBoost	High spatial detail; non-invasive; widely available	Protocol heterogeneity (thickness, kernel); limited external validation
[32,34]	PET-CT	FDG uptake, SUV metrics, textural features	Hybrid CNN + radiomics, 3D ResNet	Combines metabolic and anatomic information	Higher cost; smaller datasets; standardization issues
[30,34,36]	Histopathology/WSI	H&E slide tiles, tissue micro-architecture	Multiple-instance learning, Transformers, Graph-attention networks	Cellular-scale insight; rich morphology	Annotation burden; domain shift; interpretability concerns
[33,37]	Genomics/Transcriptomics	Mutation profiles, gene-expression panels	Random forest, DeepSurv, graph-based embeddings	Captures biological mechanisms and pathways	Small sample sizes; batch effects; integration complexity
[19,38,39]	Clinical/EMR data	Age, stage, smoking, comorbidities, labs	Logistic/Cox models, gradient-boosted trees, stacking	Good calibration; practical to deploy	Missingness; coding variability; limited linkage to images
[15,37,40]	Multimodal integration	Combined imaging + clinical + omics	Early/late fusion, ensemble meta-learners	Strongest overall discrimination and calibration; translational relevance	Requires harmonized multi-site data; higher computing and governance needs

**Table 4:** Representative AI approaches for risk and prognosis.

Reference	Approach	Typical Inputs	Example Modeling Choices	Reported Advantages
[3,34,52]	Handcrafted radiomics for malignancy risk	CT or PET/CT radiomics ± basic clinical covariates	Feature selection (e.g., LASSO) + logistic/SVM/RF/XGBoost	Better discrimination than rules; gains with light clinical fusion
[2,41,42]	Deep radiomics/CNN features	End-to-end or CNN-derived features from CT ± clinical	CNNs, hybrid deep + classical learners	Complements hand-crafted features; improved accuracy with fusion
[1,72,73]	Survival from imaging features	Radiomics or deep features ± stage/histology/treatment	Cox/penalized Cox, RSF, DeepSurv	Stronger risk stratification than clinical-only; value of longitudinal scans
[4,37,44]	Multimodal clinical-imaging fusion	CT/PET with demographics, smoking, labs, pathology, or -omics	Early/late fusion; knowledge-guided graphs	Better calibration and transportability than single-source models

**Table 5:** AI domains relevant to NSCLC staging and diagnosis.

Reference	Domain	AI Focus	Role in NSCLC Staging	Strengths/Limitations
Li et al. (2022) [79]	Radiomics	Deep Learning (CNNs)	CT/PET-based tumor & lymph-node segmentation for T/N staging	Standardizes imaging interpretation; needs large annotated datasets
Wang et al. (2019) [85]	Pathomics	Deep Learning	Tissue-level classification to distinguish invasive vs. non-invasive lesions	Improves histologic accuracy; limited pathology digitization
Wang et al. (2022) [47]	Genomics	Machine Learning	Integration of mutation and biomarker data with imaging for risk prediction	Enhances personalized staging; complex feature harmonization
Zhu et al. (2025) [53]	Immunomics	Deep Learning	Predicts immune response and metastatic potential	Aids stage-related prognosis; limited immune datasets

## Data Availability

No new data were created or analyzed in this study. Data sharing is not applicable to this article as it is a review paper based on previously published literature.
